# Preliminary investigation on the impact of salty and sugary former foods on pig liver and plasma profiles using OMICS approaches

**DOI:** 10.1038/s41598-024-70310-z

**Published:** 2024-08-21

**Authors:** Michele Manoni, Alessandra Altomare, Simona Nonnis, Giulio Ferrario, Sharon Mazzoleni, Marco Tretola, Giuseppe Bee, Gabriella Tedeschi, Giancarlo Aldini, Luciano Pinotti

**Affiliations:** 1 Department of Veterinary Medicine and Animal Science (DIVAS), University of Milan, Via dell’Università 6, 26900 Lodi, Italy; 2 Department of Pharmaceutical Sciences (DISFARM), University of Milan, Via Mangiagalli 25, 20133 Milan, Italy; 3 Agroscope, Institute for Livestock Sciences, Rte de la Tioleyre 4, 1725 Posieux, Switzerland; 4 CRC I-WE, Coordinating Research Centre: Innovation for Well-Being and Environment, University of Milan, Via Festa del Perdono 7, 20122 Milan, Italy

**Keywords:** Former food products, Pigs, Proteomics, Peptidomics, Liver, Plasma, Biotechnology, Biomarkers

## Abstract

Replacing cereals with food leftovers could reduce feed-food competition and keep nutrients and energy in the food chain. Former food products (FFPs) are industrial food leftovers no more intended for human but still suitable as alternative and sustainable feedstuffs for monogastric. In this study, omics approaches were applied to evaluate the impact of dietary FFPs on pig liver proteome and plasma peptidome. Thirty-six Swiss Large White male castrated pigs were randomly assigned to three dietary treatments [control (CTR), 30% CTR replaced with salty FFP (SA), 30% CTR replaced with sugary FFP (SU)] from the start of the growing phase (22.4 ± 1.7 kg) until slaughtering (110 ± 3 kg). The low number of differentially regulated proteins in each comparison matrix (SA/SU vs. CTR) and the lack of metabolic interaction indicated a marginal impact on hepatic lipid metabolism. The plasma peptidomics investigation showed low variability between the peptidome of the three dietary groups and identified three possible bioactive peptides in the SA group associated with anti-hypertension and vascular homeostasis regulation. To conclude, the limited modulation of liver proteome and plasma peptidome by the SA and SU diets strenghtened the idea of reusing FFPs as feed ingredients to make pig production more sustainable.

## Introduction

The higher the demand for animal source food, the higher the use of cereal crops for pig nutrition, exacerbating feed-food competition^1^. The research for alternative and sustainable feedstuffs could decrease the environmental impact of pig production^2,3^. Food leftovers have a lower environmental impact than the original product but are still nutritionally valid^4^. Former food products (FFPs) are food leftovers dropping out from the production of ready-to-eat products such as snacks, chips, biscuits, and bread, originally intended for human consumption but no more suitable for that due to technical or logistical defects^1^. Also, since FFPs originate from processed and ultra-processed food, they contain compounds from specific food ingredients such as cocoa, coffee, and chocolate^5^.

The FFPs were used to partially replace conventional cereals for post-weaning piglets, growing, and finishing pigs because of the high energy, simple sugars and highly-digestible starch content of FFPs^6^. This is a sustainable way to recycle food leftovers and keep their nutritional value in the feed-food chain. The FFPs were used as blend^7,8^ or divided into salty FFPs (SA) and sugary FFPs (SU)^9^. The SA and SU FFPs were formulated to be isoenergetic but evaluated separately to discriminate the effect of their ingredients on the animal. Replacing conventional cereals up to 30% with FFPs as blend did not impair the growth performance of post-weaning piglets^7,8^. Further research was conducted by using SA and SU FFPs diets, and neither dietary treatments used to replace conventional cereals up to 30% altered the growth performance and the feeding behaviour of post-weaning piglets^9^. Interestingly, a higher level of coffee- and cocoa-related metabolites, such as caffeine and theobromine, was observed in the plasma of post-weaning piglets fed FFPs diets^10^, as similarly observed in humans^11^.

This study follows previous work of Mazzoleni et al.^12^ and Tretola et al.^13^, in which 30% of SA and SU FFPs were included into adult pig diets to replace conventional cereals. The FFPs diets did not impair the growth performance, feeding behaviour and carcass composition of adult pigs^12^, did not affect any meat sensory trait (i.e. pH, temperature, water holding capacity, shear force, and colour) and did not lead to intramuscular fat accumulation in meat, despite slight variation in the fatty acid profile of backfat, mostly related to the fatty acid profile of the diets^13^. Furthermore, no significant alteration was observed in the level of a panel of serum metabolites related to liver disease and hepatic fatty acids accumulation^13^, such as alanine aminotransferase, aspartate aminotransferase, alkaline phosphatase, total bilirubin and cholesterol, albumin, and globulins^12^. Specifically, albumin is known to be one of the most abundant circulating proteins with antioxidant activity by trapping free radicals^14^, and the level of which was not altered in the plasma of pigs receiving FFPs diets^12^. In light of these results that confirmed the potential of FFPs as alternative and safe feed ingredients, this study was aimed at further increasing the knowledge on FFPs by evaluating the potential impact of the FFPs diets on the metabolism of pigs. First, the liver was targeted because it is the major metabolic centre in the organism involved in body energy regulation and metabolism of nutrients and exogenous compounds. The effect of the diet is reflected on the hepatic pathways involving amino acids^15^ and fatty acids^16,17^. Then, plasma was targeted to observe the impact of experimental diets on the physiology of the organism^18^. Therefore, we decided to apply shotgun label-free quantitative proteomics and peptidomics to investigate how the dietary inclusion of SA and SU FFPs could affect the pig physiology in terms of liver proteome and plasma peptidome profiles. So far, proteomics was rarely applied to evaluate alternative feedstuffs for pigs. Instead, peptidomics is a tool often used for identifying diet-related biomarkers or for detecting bioactive peptides^19,20^. The coupling of these two omics approaches is considered an opportunity to enlarge the amount of data available and to obtain a broader picture of the metabolic and physiological state of the animal^21,22^.

The digestion rate of starch is known to affect the regulation of blood glucose and insulin levels and the lipid metabolism in liver^23,24^. Then, the amount of dietary fat content is related to altered liver functions, such as liver lipid accumulation. In growing pigs fed a high-fat diet (301 vs. 44 g/kg dry matter (DM) of standard diet), there was an increased level of serum lipids but no evidence of liver lipid accumulation, while the proteins involved in fatty acid oxidation were upregulated^25^. In addition, the fatty acid profile affects liver lipid metabolism. Pigs at 60 kg body weight (BW) were fed until slaughtering (100 kg BW) with diets containing seven dietary oil and fat sources. The authors observed that several lipogenic genes in liver were downregulated by fish oil inclusion (high poly-unsaturated fatty acids (PUFA) content) but upregulated by high-oleic sunflower oil (high mono-unsaturated fatty acids (MUFA) content), suggesting the central role of fatty acid saturation in the regulation of liver lipogenesis in pigs^26^. Also, caffeine and theobromine are biotransformed in liver, and a stimulating effect on hepatic lipid oxidation together with a reduced expression of lipogenic genes (lowering the risk of non-alcoholic fatty liver disease) was observed in mice after caffeine^27^ and theobromine^28^ supplementation. However, theobromine was considered an undesirable compound in feed^5^, therefore its biotransformation along with its effect on hepatic lipid metabolism must be further addressed.

Concomitantly, the plasma peptidome of the same animals was evaluated to disclose if SA and SU diets could modulate the peptidomic profile with a particular focus on possible bioactive peptides. It is well known that various raw food materials contain bioactive peptides that exert a specific physiological function in tissues and fluids of living organisms^29–32^. In parallel, gastrointestinal digestion of dietary proteins generates a huge number of peptides that become bioactive once released by proteolysis in the gut^33,34^. Peptidomics approaches have been introduced recently in research concerning animal feeding. However, while dietary exogenous proteins and peptides released in the gut during digestion have been investigated extensively, the possibility that non-dietary proteins that are also present in the digestive tract may give rise to endogenous bioactive peptides has only recently received attention^35^. In a study conducted on pigs fed a casein-based diet, up to 80% of endogenous proteins was digested and reabsorbed by the end of the small intestine into the bloodstream^36^. Moreover, Dave et al.^37^ demonstrated that large numbers of bioactive peptides exist within the amino acid sequences of endogenous proteins, and they may be cleavable by digestive enzymes. Therefore, it is likely that these proteins, as well as dietary proteins, can be a source of latent bioactive peptides (from 2 to greater than 40 amino acids long) that, when released during digestion in the gastrointestinal tract, can act as modulators of various physiological functions^38–40^. Despite the biological activity of these endogenous peptides is still little studied, there is evidence that some of these peptides possess a range of effects including antihypertensive, cholesterol-lowering, antioxidant, anticancer, immunomodulatory, antimicrobial, opioid, antiobesity, and mineral binding effects^40^.

In this study, label-free mass spectrometry-based omics approaches were applied to the liver proteome and plasma peptidome of pigs fed diets with 30% dietary inclusion of SA and SU FFPs to replace conventional cereals. The aim was to observe how FFPs modulate the liver proteome and related metabolic pathways and to identify all endogenous plasma peptides, with particular reference to the corresponding bioactive ones.

## Results

### Hepatic protein profile

The reproducibility of the biological and technical replicates was confirmed by the calculation of Pearson’s linear correlation factor, which exceeded 0.98 for all experimental conditions, compared in pairs (Supplementary Fig. S1).

From a technical point of view, the proteomic analysis allowed for the identification and quantification of 2881 proteins by merging all experimental conditions. Then, for each observation, the extent of the protein abundance variation (log2 FC) was evaluated (Table 1).

**Table 1:** Technical parameters of proteomics quantification.

	SA versus CTR	SU versus CTR	SA versus SU
Significantly modulated proteins (n)	54	47	24
Log2 Fold-change > 0.5 (n)	9	14	5
Log2 Fold-change < -0.5 (n)	11	10	7

For each comparison matrix, the number of significantly quantified proteins and the number of significantly modulated proteins, both in terms of up-regulation and down-regulation, were reported. Proteins were considered significantly modulated if *P* < 0.05.

**Figure 1:**
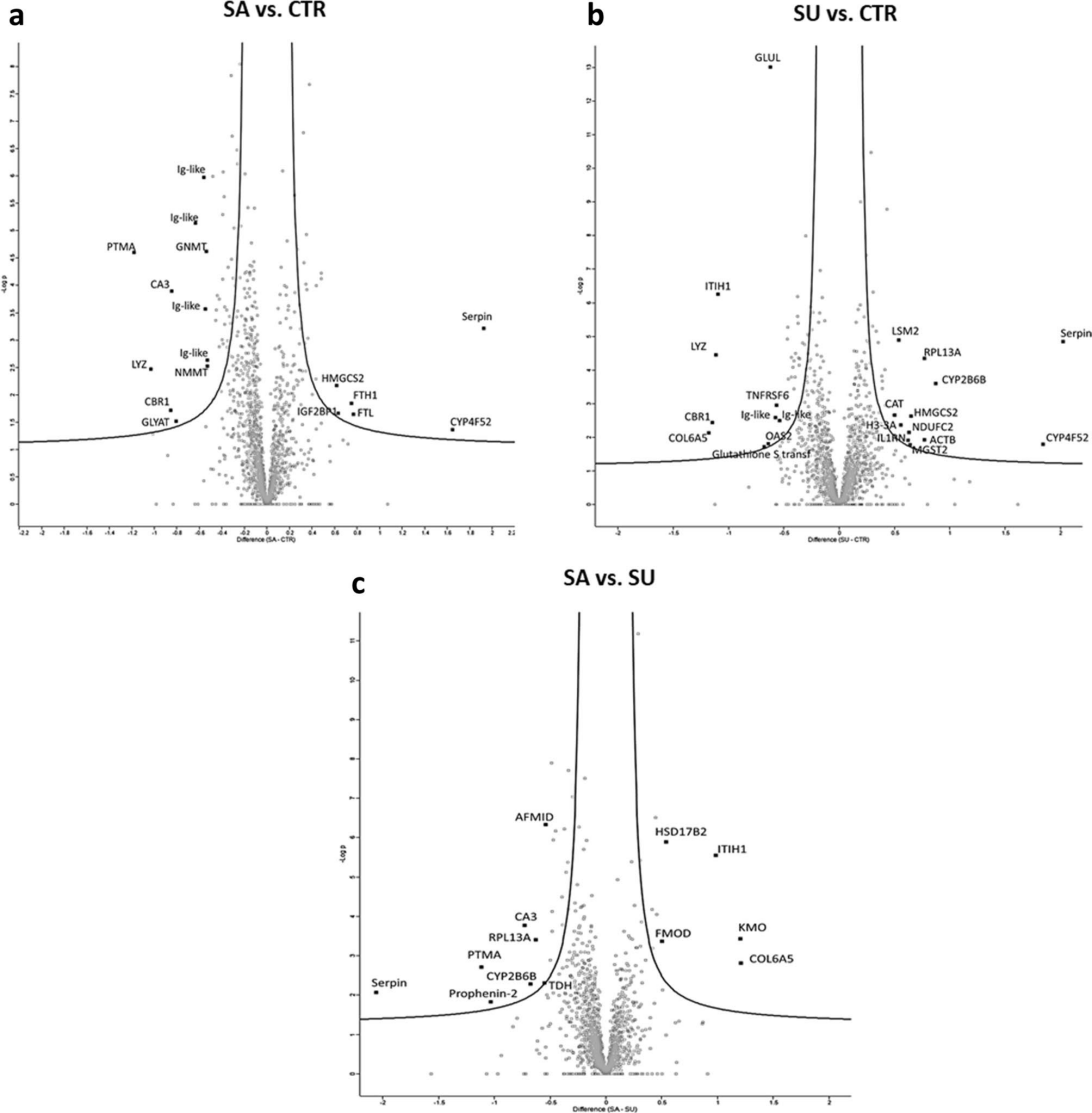
Volcano Plots derived from the LFQ analyses of SA versus CTR (**a**), SU versus CTR (**b**), SA versus SU (**c**) comparison matrices.

Figure 1 shows the Volcano Plots of the distribution of log2 FC values versus -LogP values, indicative of the significance of the calculation, where significantly up-regulated gene products are on the right side and significantly down-regulated ones on the left side of the graph.

A total of 125 proteins was significantly modulated considering the three comparison matrices (SA vs. CTR, SU vs. CTR, and SA vs. SU). Of this, 28 proteins were upregulated (log2 FC > 0.5) and 28 were downregulated (log2 FC < − 0.5). Functional enrichment of significantly modulated proteins and thus gene annotation-based interactome analysis were performed. In this case, the String software was used for searching potential interactions among significantly up-regulated and significantly down-regulated proteins. The results showed that the minimal modulation exerted by the SA and SU dietary treatments led to no correlation between the modulated proteins. Despite this result, a further data exploration was done manually, investigating individual proteins to obtain a picture of the most interesting biological information that emerged from the proteomics results and the literature.

### Proteome modulation induced by the salty diet (SA vs. CTR)

Table 2 summarizes the most relevant significantly modulated gene products in the SA group compared to the CTR group, together with the log2 FC values and a panel of values indicating the animal reproducibility, namely the number of animals confirming each evidence (if the value was confirmed in ≥ 2 technical replicates, the biological replicate was considered valid; identifications absent in the pool and in less or equal to 3 biological replicates were excluded).

**Table 2:** Most relevant significantly modulated proteins in the SA versus CTR comparison matrix.

Quantitative analysis	Functional evaluation		Animal reproducibility	
Log2 FC	Gene name	Protein name	SA	CTR
− 1.031	LYZ	Lysozyme C-3	4	5
− 0.970	CA3	Carbonic anhydrase 3	7	7
− 0.804	GLYAT	Glycine *N*-acyltransferase-like protein	7	7
− 0.659	*UniProtKB unreviewed (TrEMBL)*	*Ig-like domain-containing protein*	7	7
− 0.651	*UniProtKB unreviewed (TrEMBL)*	*Ig-like domain-containing protein*	7	7
− 0.573	NNMT	Nicotinamide *N*-methyltransferase	7	6
− 0.572	LOC100156325	Serpin domain-containing protein	6	5
0.616	HMGCS2	Hydroxymethylglutaryl-CoA synthase, mitochondrial	7	7
0.716	GSTA4	Glutathione transferase	5	4
0.765	FTL	Ferritin	7	7
0.953	H3-3A	Histone H3	3	3
0.991	FTH1	Ferritin	7	7
1.278	H3-C1	Histone H2A/H2B/H3 domain-containing protein	6	5

Negative values of log2 FC indicate the corresponding gene products’ down-regulation, while positive values the gene products’ up-regulation. The “animal reproducibility” columns indicate the number of animals per experimental group that confirms the evidence (up/down-regulation).

Among the most interesting findings regarding lipid metabolism there are the downregulation of carbonic anhydrase (CA3) and nicotinamide N-methyltransferase (NNMT), and the up-regulation of mitochondrial hydroxymethylglutaryl-CoA synthase (HMGCS2) in the SA group compared to the CTR group. Then, other upregulated gene products of interest were also FTL and FTH1, which encode for the light chain and heavy chain of the ferritin protein, respectively, and glutathione transferase GSTA4, which conjugates glutathione to numerous endogenous and exogenous electrophiles. The last but not the least important evidence concerned the upregulation of histone proteins (H3-3A and H3-C1).

### Proteome modulation induced by the sugary diet (SU vs. CTR)

Table 3 summarizes the most relevant significantly modulated gene products following SU diet feeding compared to the CTR, along with log2 FC values and a panel of values indicating the reproducibility of the animals.

**Table 3:** Most relevant significantly modulated proteins in the SU versus CTR comparison matrix.

Quantitative analysis	Functional evaluation		Animal reproducibility	
Log2 FC	Gene name	Protein description	SU	CTR
− 1.659	COL6A5	Collagen type VI alpha 5 chain	5	7
− 1.244	LYZ	Lysozyme C-3	5	5
− 0.970	ITIH1	Inter-alpha-trypsin inhibitor heavy chain 1	7	7
− 0.728	TNFRSF6	FAS-associated death domain protein	5	4
− 0.675	LOC100526118	Glutathione S-transferase	6	6
− 0.652	GLUL	Glutamine synthetase	7	7
− 0.642	OAS2	2′-5′-Oligoadenylate Synthetase 2	6	5
− 0.641	FMO4	Flavin-containing monooxygenase	4	5
− 0.573	UniProtKB unreviewed (TrEMBL)	Ig-like domain-containing protein	6	7
0.575	LSM2	U6 snRNA-associated Sm-like protein LSm2	4	4
0.630	NDUFC2	NADH dehydrogenase [ubiquinone] 1 subunit C2	3	3
0.648	HMGCS2	Hydroxymethylglutaryl-CoA synthase, mitochondrial	7	7
0.724	GRK3	G protein-coupled receptor kinase	3	3
0.769	ACTB	Actin B	6	7
1.012	H3-3A	Histone H3	6	5

Negative values of log2 FC indicate the corresponding gene products’ down-regulation, while positive values the gene products’ up-regulation. The “animal reproducibility” column indicates the number of animals per experimental group that confirms the evidence (up/down-regulation).

Here, the trend of HMGCS2 upregulation induced by the SU diet compared to CTR was superimposable to that observed for the SA, whereas no other gene product related to lipid metabolism was observed. In addition, as shown for the SA versus CTR comparison, the upregulation of histone protein H3 was also significant for the SU diet. Then, the upregulation of actin together with the downregulation of collagen type 6 (alpha chain 5) and inter-alpha-trypsin inhibitor heavy chain 1 (ITIH1) were observed in the SU group compare to CTR.

### Proteome modulation induced by the salty diet compared to the sugary diet (SA vs. SU)

Finally, Table 4 summarizes the most relevant gene products found to be significantly modulated by comparing the intake of the SA diet with the SU diet. The purpose was to confirm the experimental evidence already provided by the corresponding comparison matrices compared to the CTR.

**Table 4:** Most relevant significantly modulated proteins in the SA versus SU comparison matrix.

Quantitative analysis	Functional evaluation		Animal reproducibility	
Log2 FC	Gene name	Protein description	SA	SU
− 2.119	LOC106504547	Serpin domain-containing protein	5	4
− 0.810	CYP2B6B	Unspecific monooxygenase	3	3
− 0.776	CA3	Carbonic anhydrase 3	7	7
− 0.752	TDH	L-threonine 3-dehydrogenase, mitochondrial	7	7
− 0.631	RPL13A	60S ribosomal protein L13a	4	3
0.644	FAH	Fumarylacetoacetase	7	7
1.020	ITIH1	Inter-alpha-trypsin inhibitor heavy chain 1	7	7
1.465	KMO	Kynurenine 3-monooxygenase	4	3
1.644	COL6A5	Collagen type VI alpha 5 chain	5	4

Negative values of log2 FC indicate the corresponding gene products’ down-regulation, while positive values the gene products’ up-regulation. The “animal reproducibility” column indicates the number of animals per experimental group that confirms the evidence (up/down-regulation).

The analysis confirmed that the downregulation of CA3 was specific to the SA diet, as well as the downregulation of ITIH1 and COL6A5 upon the SU diet intake confirmed to be specific for the SU diet. The absence of modulation of HMGCS2 and H3-3A also confirmed their dependence on the intake of both SA and SU diets compared to CTR. Interestingly, the SA diet mediated the upregulation of the Kynurenine 3-monooxygenase (KMO), whose modulation was not significant in the SA versus CTR comparison, but in this case the significant upregulation indicated that the SA diet had a higher KMO level compared to the SU diet. Therefore, the SU diet might have a lower KMO level than the CTR diet, otherwise the KMO level should have been significantly different also in the case of the SA versus CTR comparison. The Supplementary Figure S2 shows this hypothetical comparison of the KMO level in the three dietary groups considering the level of significance.

### Plasma peptidomic profile

Considering as positively identified all the peptides present in at least 70% of each data set, 84 (Additional file: Additional Table 1), 98 (Additional file: Additional Table 2) and 89 peptides (Additional file: Additional Table 3) were detected in CTR, SA and SU group, respectively, for a total of 122 identified peptides (Additional file: Additional Table 4).

### Evaluation of the intra-group variability and comparison of the plasma peptidome

The individual variability of the biological replicates, evaluated by comparing plasma samples belonging to the same experimental group in terms of number of identified peptides and sum of LFQ signal intensity demonstrated a very low variability, most often less than 10% (Supplementary Table S1).

**Figure 2:**
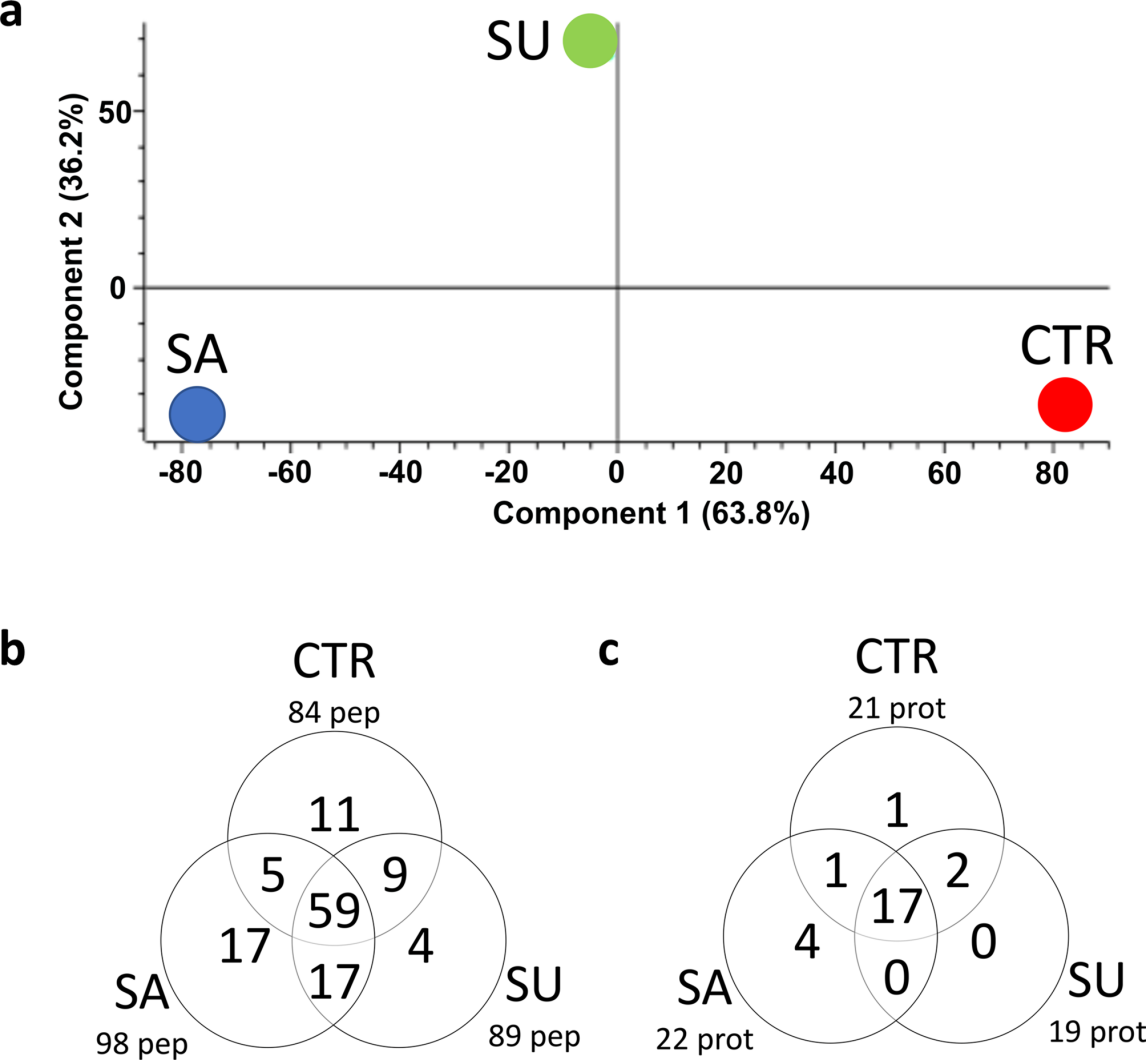
Schematic representation of peptidomic results. Legend: PCA analysis of the peptidome after grouping and averaging quantitative data related to peptides in CTR (red), SA (blue) and SU (green) group (**a**), Venn diagram of all peptides identified in plasma samples from the comparison CTR versus SA versus SU (**b**), and Venn diagram of all the proteins of which produced by the proteolysis that generated peptides identified in plasma samples from the comparison CTR versus SA versus SU (**c**).

The Pearson’s correlation coefficient values (Additional file: Additional Table 5A–C) confirmed the strong correlation between datasets of the same group without any outlier sample. A principal component analysis (PCA) was carried out on the peptidome of all thirty-six samples, suggesting a differential clustering of the three groups (Fig. 2A).

The three-way comparison between the peptidome of plasma samples obtained from pigs of all the three dietary groups allowed to identify peptides in common and those that were exclusively present in one group. In particular, 59 peptides were present in all groups (Additional file: Additional table 6), 11 were exclusively identified in CTR (Additional file: Additional table 7), 17 in SA (Additional file: Additional table 8) and 4 in SU group (Additional file: Additional table 9) (Fig. 2B).

All these peptides were produced by the proteolysis of 25 plasma proteins (Additional file: Additional table 10) (Fig. 2C), of which 68% (n = 17) common to CTR, SA and SU plasma. Only an uncharacterized protein was exclusively identified in CTR and 4 proteins in SA group, namely: heparan sulfate proteoglycan 2 (HSPG2), poly(A) binding protein cytoplasmic 4 (PABPC4), vanin 3 (VNN3) and legumain (LGMN).

### Search for bioactive peptides

The search of potentially bioactive peptides was carried out in SATPdb on all data sets from CTR, SA and SU groups. Interestingly, three peptides with antihypertensive activity were exclusively identified in the SA group (Table 5).

**Table 5:** List of potentially anti-hypertensive peptides found in plasma samples from SA group.

Database SATPdb sequence	Sequence identified in SA peptides	Protein of origin	Gene
LSLP	LRAYDG**LSLP**EDAETISAGRAG	A0A286ZHV7	HSPG2
RALP	NQYMQRVAGM**RALP**ANAILNQFQ	A0A8W4FKU3	PABPC4
VFER	ALYGR**VFER**DPPRLGQGPGQVQ	F1S3Q8	VNN3

In bold the sequence stretches corresponding to the SATPdb peptides with anti-hypertensive activity.

According to the SATPdb database, the peptides LRAYDGLSLPEDAETISAGRAG and ALYGRVFERDPPRLGQGPGQVQ contained a sequence stretch corresponding to the SATPdb peptides with anti-hypertensive activity. They originated from the proteins heparan sulfate proteoglycan 2 (HSPG2) and vanin-3 (VNN3), respectively. In addition, the peptide NQYMQRVAGMRALPANAILNQFQ originated from cytoplasmic poly(A) binding protein 4 (PABPC4).

**Figure 3:**
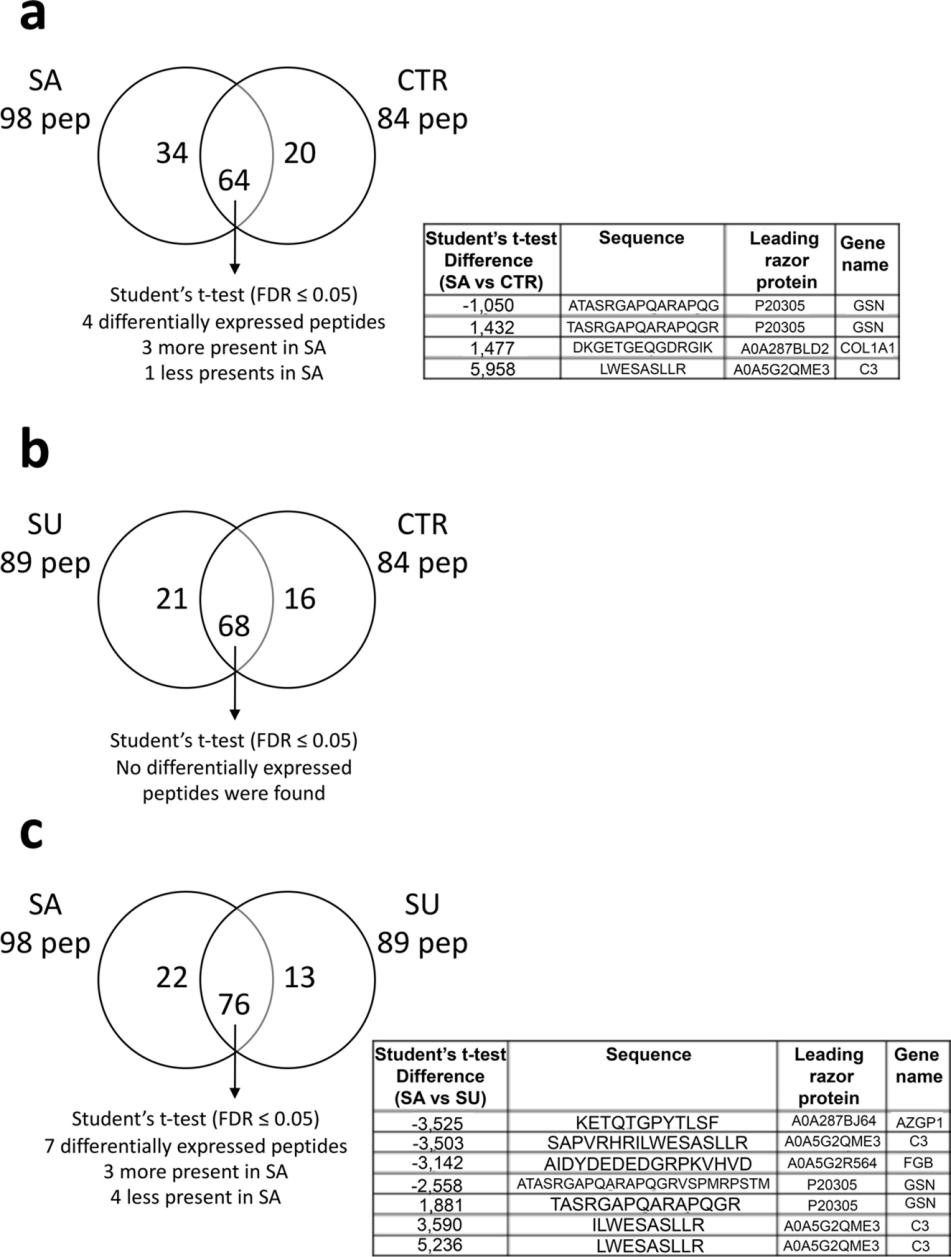
Schematic representation of the peptidomics two-way comparisons results. Venn diagram of all peptides identified in plasma samples from the comparison SA versus CTR (**a**), SU versus CTR (**b**), and SA versus SU (**c**). Tables report the list of peptides that showed significant t-test difference (FDR ≤ 0.05).

Specific analyses, carried out by two-way comparisons between the peptidome of plasma samples obtained from pigs fed with three different diets (SA vs. CTR, SU vs. CTR, and SA vs. SU), allowed to identify peptides in common and those that were exclusively present in one group. A Student’s t-test (FDR ≤ 0.05) was carried out to identify peptides differentially present among the different conditions. Peptides were significantly different if they were identified only in one condition or showed significant t-test difference (FDR ≤ 0.05) (Fig. 3). According to our results, 20 and 16 peptides were exclusively expressed in CTR in the comparisons SA versus CTR and SU versus CTR (respectively), then 34 and 22 peptides were exclusively expressed in SA in the comparisons SA versus CTR, and SA versus SU (respectively), whereas 21 and 13 peptides were exclusively expressed in SU in the comparisons SU versus CTR, and SA versus SU (respectively). However, no potentially bioactive peptides were identified by searching in SATPdb and DFBP database on peptides that showed significant t-test difference (FDR ≤ 0.05), according to the results described above.

## Discussion

This study followed previous research about the use of FFPs as alternative and sustainable feedstuffs for pigs. After observing that SA and SU FFPs diets were not detrimental for the growth and feeding behavior of piglets and pigs, we decided to apply omics approaches to further evaluate the impact of FFPs by focusing on liver proteome and plasma peptidome.

A label-free quantitative proteomics strategy was applied on liver tissue samples with the aim of assessing the impact of the SA and SU diets on the modulation of liver function and of any relevant cellular pathway. Specifically, the experimental conditions tested were compared with each other in order to obtain a comprehensive picture elucidating the extent of liver proteome modulation by the salty diet (SA vs. CTR), as well as by the sugary diet (SU vs. CTR) and by comparing the two experimental diets (SA vs. SU). As seen above, the results of the functional analysis only showed a minimal modulation exerted by the SA and SU diets on liver proteome and any significant correlation between the modulated proteins was detected. However, we decided to further explore the proteomics results by observing and discussing the biological role of the most relevant modulated proteins.

The SA versus CTR comparison (Table 2) and the SU versus CTR comparison (Table 3) pointed out the modulation of several proteins involved in hepatic lipid metabolism. Compared to CTR group, the SA diet led to the downregulation of CA3 and NNMT and the up-regulation of HMGCS2, whereas the SU diet only caused the upregulation of HMGCS2 (similarly to the SA diet). The CA3 protein catalyzes the reversible CO_2_ hydration/dehydration reaction, which is necessary for the synthesis of long chain fatty acids, requiring bicarbonate. Nicotinamide *N*-methyltransferase (NNMT) catalyzes the methylation of nicotinamide and it is involved in hepatic lipid metabolism and energy homeostasis^41^. Besides, HMGCS2 is the rate-limiting enzyme in the ketogenesis pathway (a biochemical process by which organisms produce ketone bodies by cleaving fatty acids and ketogenic amino acids). A dysregulated ketogenesis is associated with fatty liver development^42^. Such differentially expressed proteins might suggest an altered lipid metabolic activity induced by the higher crude fat content and the different fatty acid profile of the SA and SU diets compared to the CTR diet, in particular a higher MUFA content for both SA and SU diets and a lower SFA content for the SA diet compared to CTR^12^. The suggested alterations in lipogenesis and lipolysis pathways in liver could be related to the fact that the SA and SU FFPs diets affected the synthesis of fatty acids and the related fatty acid profile of adipose tissue^43^, although the level of intramuscular fat in meat and the major meat sensory traits were not altered by both FFPs diets^13^. Indeed, Luciano et al.^43^ reported that FFPs were able to affect the fatty acid profile of subcutaneous adipose tissue in piglets. The main effects exerted by SA and SU FFPs was to increase the proportion of MUFA in FFPs-fed piglets compared to the control group. For PUFA, the SU FFPs group showed the lowest value compared to the other groups. A similar trend was also cofimed by Tretola et al.^13^, thus confirming that the fatty acid composition of the diet influences that of the adipose tissue in pigs^44^.

The upregulation of NNMT was responsible for PPARγ transactivation and related lipid accumulation in a model of mouse liver cells challenged with palmitic acid, a SFA known to be positively correlated to hepatic lipotoxicity^45^. The low SFA content of the SA diet could be related to the downregulation of NNMT, whereas the higher MUFA content of both SA and SU diets could be related to the upregulation of HMGCS2. To summarize, we hypothesized that the downregulated CA3 reduced lipogenesis and that lower NNMT level and the upregulated HMGCS2 led to reduced lipid accumulation in liver. However, the suggested perturbation of hepatic lipid metabolism did not significantly affect the lipid content of pork meat from a quantitative point of view, although an effect of the diets on the fatty acid profile of backfat but not of intramuscular fat was observable^13^.

In addition, the upregulation of FTL, FTH1, and GSTA4 in the SA group was observed (Table 2). The FTL and FTH1 proteins are both involved in the storage of iron in a non-toxic form. The heavy chain (FTH1) also possesses ferroxidase activity^46^. The glutathione transferase GSTA4 and in general GST genes are known to be upregulated in response to oxidative stress^47^. If the upregulation of proteins involved in iron storage could be related to increased dietary iron intake, the overexpression of GSTA4 could indicate a cellular response to oxidative stress. Instead, glutathione S-transferase was found to be downregulated by the SU diet (Table 3), suggesting that the primary antioxidant defense was activated to an even lesser extent than in the CTR condition. However, in the present study any marker of oxidative stress was measured.

Then, both SA and SU diets led to the upregulation of histone proteins compared to CTR (Tables 2 and 3), which are considered an early response to cellular stress and occurred before the release of markers of stress including heat shock proteins. The post-translational modifications of histone H3 work as epigenetic regulators of gene transcription, influencing chromatin structure and providing binding sites for many transcription factors, thereby regulating various cellular functions, such as gene expression, cell cycle, replication, and stress-induced DNA repair. However, the upregulation of histone proteins was not correlated with any metabolic pathway involved in cellular stress. In the SU group, a hypothetical reorganization of the cell structure could be suggested by the modulation of actin, collagen type 6 (alpha chain 5) and inter-alpha-trypsin inhibitor heavy chain 1 (ITIH1) (Table 3), which is mainly involved in stabilizing the extracellular matrix by binding with hyaluronic acid.

Finally, the upregulation of KMO, a mitochondrial enzyme involved in the kynurenine pathway (KP) of the tryptophan degradation, was observed in the SA group compared to SU group (Table 4). The KMO controls the synthesis of several kynurenine metabolites, including 3-hydroxykynurenine (3-HK), quinolinic acid (QUIN) and kynurenicacid (KYNA), as well as anthranilic acid. These bioactive metabolites are associated with inflammatory conditions^48^. A positive correlation was observed between the increased hepatic fatty acid oxidation and tryptophan metabolites concentration, such as the KMO-mediated kynurenine metabolites cited above, in rat serum^49^. Here, we hypothesized that HMGCS2 upregulation mediated by both SA and SU diets was correlated with reduced lipid accumulation in liver, but the SA diet also led to CA3 and NNMT downregulation. Therefore, the alteration of lipid metabolism caused by the SA diet might explain the significant upregulation of KMO compared to the SU diet, although no measurement of kynurenine metabolites was performed.

The peptidomics analysis showed low intra-group variability (around 10% in all cases) (Supplementary Table S1), whereas the PCA showed a differential clustering of the CTR, SA, and SU groups. Around 70% of plasma proteins from which the detected peptides were derived was shared by the three dietary groups, but four proteins were specific of the SA group. Heparan sulfate proteoglycan 2 (HSPG2) is a core protein to which three long chains of glycosaminoglycans are attached. This protein binds to and cross-links many extracellular matrix components and cell-surface molecules, as laminin, prolargin, collagen type IV, and transthyretin, playing an essential role in multiple biological activities, such as helping to maintain the endothelial barrier function. It is a potent inhibitor of smooth muscle cell proliferation and is thus thought to help maintain vascular homeostasis^50^. Vanin genes (VNN) are clustered and encode isoforms of pantetheinase, whicho hidrolyzes pantetheine into pantothenic acid (vitamin B5), and cysteamine (CysH, 2-aminoethanethiol), a sulfhydryl compound which is in equilibrium with its oxidized form cystamine (CysN). The role of vanins in the regulation of mucosal inflammation may contribute to structural damage of the intestinal mucosa^51,52^. In particular, vanin-1 and vanin-2 are ectoenzymes (GPI)-linked anchored to the cell surface, expressed by different organs among which the intestinal tract. They have pantetheinase enzymatic activity, but the activity of vanin-2 is weaker than that of vanin-1. Vanin-3, which generated a peptide exclusively identified in SA group, by lacking the GPI-anchoring consensus, encodes a secreted truncated protein whose expression is induced by oxidative stress^53^. Legumains (LGMN) are cysteine protease with a receptor modulating effect on integrin αvβ3 that alters the downstream signaling cascades in vascular smooth muscle cells^54^. Also in this case, the presence of peptides originated by its degradation may be relatable with the SA diet since this endogenous modulator of integrin triggers vascular degeneration, dissection, and rupture.

Altogether, the peptidomics analysis did not highlight significant differences between the three diets, substantially confirming what was highlighted by the proteomic analysis with the only exception of the four proteins exclusively identified in the SA group, whose described activity was related to keep vascular homeostasis and counteract inflammation, oxidative stress and vascular degeneration.

Of the three peptides exclusively identified in the SA group (Table 5), the peptides LRAYDGLSLPEDAETISAGRAG and ALYGRVFERDPPRLGQGPGQVQ originated from the proteins HSPG2 and VNN3, respectively, whose involvement in helping maintain vascular homeostasis and in oxidative stress response was discussed above. In addition, the peptide NQYMQRVAGMRALPANAILNQFQ originated from the protein PABPC4, whose involvement in the anti-hypertensive response is not reported in the literature, but possesses the RALP sequence whose anti-hypertensive and renin-inhibitory activities are described in both the SATPdb and the DFBP database. Altogether, the peptidomic results suggested that the SA diet, with about twofold higher sodium content compared to CTR and SU diets (3.2 g/kg DM in SA diet versus 1.7 g/kg DM in both SU and CTR diets)^12^, could have triggered the activation of adaptive physiological mechanisms through which pigs tried to counteract any damage that could have been caused by the content of sodium in the SA diet, generating potentially anti-hypertensive plasma peptides from proteins involved in helping maintain vascular homeostasis and oxidative stress response. The presence of an increased number of peptides with biological activity in the plasma of SA-fed pigs was interesting, even though the bioavailability of peptides has been often questioned^55,56^.

To summarize, the proteomics investigation identified a low number of differentially modulated proteins with no metabolic interaction between them, indicating a limited impact on liver function. The literature investigation showed a partial modulation of hepatic lipid metabolism by SA and SU diets, with reduced lipogenesis and increased lipid oxidation. Both SA and SU diets induced dysregulation of histone protein H3, attributable to a possible remodulation of cellular structure. In any case, these results indicated that both SA and SU diets did not deeply affect the liver function. Then, the peptidomics investigation identified no relevant differences between the three dietary groups, with the only exception of the three endogenous peptides exclusively identified in the SA group and related to antihypertensive activity and regulation of vascular homeostasis. In conclusion, the metabolic impact of SA and SU FFPs diets in pigs was limited and very few liver proteins and plasma peptides were differentially regulated by SA and SU diets. These findings provided further evidence on the validity of reusing FFPs in pig nutrition, to keep their nutritional value in the food chain and reduce the feed-food competition.

It is important to note that this study is subject to potential limitations. Primarily, our proteomic investigation considered the modulation of the hepatic proteome and the metabolic impact of the altered proteins, but did not validate the modulation of individual hepatic proteins. Due to the relatively small sample size of the present study, we formulated hypotheses that should be the subject of further research in the future. For example, the application of immunometric assays to verify our results on the modulation of individual hepatic proteins could provide a more accurate assessment of the potential effect of the experimental diets on the modulation of hepatic lipid metabolism. Furthermore, the bioavailability and bioactivity of the three SA-related peptides have not been determined and require additional validation studies. Consequently, further research is necessary to validate these hypotheses and confirm or challenge our findings.

## Methods

### Reagents

Purified and sterilized bi-distilled water with the Milli-Q™ system (Millipore, Bedford, MA, USA) was used to prepare the buffers or for other applications that required it. The following reagents were used for tissue homogenisation: SDS (sodium dodecyl sulphate), supplied by Bio-Rad (Hercules, CA, USA), NaCl (sodium chloride) and MgCl_2_ (magnesium chloride), from Fluka Chemical (Ronkonkoma, NY, USA), TEAB (triethylammonium bromide) and Benzonase® Nuclease, purchased from Sigma-Aldrich (Milan, Italy) and, finally, cOmplete™ protease inhibitor cocktail, purchased from Roche Diagnostics GmbH (Mannheim, Germany). The protein concentration was performed using the BCA kit (Sigma-Aldrich, Milan, Italy).

For the proteolytic digestion of protein extracts, solutions containing TCEP (tris(2-carboxyethyl) phosphine), IAA (iodoacetamide) and AMBIC (ammonium bicarbonate) supplied by the company Sigma-Aldrich (Milan, Italy) were prepared; other reagents used during proteolytic digestion were: H_3_PO_4_ (phosphoric acid, Fluka Chemical, Ronkonkoma, NY, USA), MeOH (methanol, Sigma-Aldrich, Milan, Italy) and TEAB (see above), while trypsin (sequencing-grade trypsin, Roche, Mannheim, Germany) was chosen as the proteolytic enzyme. S-TRAP spin-columns, purchased from Protifi (Huntington, NY, USA), were used for maximising efficiency of protein digestion. The ultrapure formic acid (FA) and acetonitrile (ACN) used in the LC–MS analysis were supplied by Sigma-Aldrich™ (Milan, Italy), as well as Amicon Ultra-0.5 mL centrifugal filters (MWCO 10K) and trifluoroacetic acid (TFA) used for the high molecular weight protein depletion in peptidomics. Plasma samples cleanup, recovery, and concentration were performed using Zip-Tip C18 Millipore (Billerica, MA, USA).

### Experimental design

The in vivo trial complied with the ARRIVE guidelines and was authorized by the Swiss Federal Committee for Animal Care and Use (authorization number: 2021-35-FR). The in vivo trial was performed at the Agroscope research unit located in Posieux, Switzerland. All methods were performed in accordance with relevant guidelines and regulations. Thirty-six Swiss Large White male castrated pigs (22.4 ± 1.7 kg initial BW) were assigned to three isoenergetic and isonitrogenous dietary treatments at the beginning of the growing period: 1) control diet (conventional cereals, CTR), 2) replacement of 30% CTR with salty FFPs (SA), and 3) replacement of 30% CTR with sugary FFPs (SU). The dietary treatments lasted until the pigs reached the required BW for slaughtering (110 ± 3 kg final BW)^12^. The diets were characterized by Mazzoleni et al.^12^. Briefly, the three diets in the finishing phase had a similar content of crude protein and crude fibre. The SA diet had approximately double the sodium content compared to the SU and CTR diets, whereas the SU diet had a higher crude fat content (59.0 g/kg DM) than SA diet (53.0 g/kg DM) and CTR diet (45.0 g/kg DM). The SU diet had a higher saturated fatty acids (SFA) content (20.0 g/kg DM) than SA diet (12.0 g/kg DM) and CTR diet (16.0 g/kg DM), and the SA diet had a higher MUFA content (29.0 g/kg DM) compared to SU diet (25.0 g/kg DM) and CTR diet (14.0 g/kg DM). Instead, the content of PUFA was similar for all the diets.

### Slaughter procedure and liver and plasma sampling

The pigs were slaughtered at the Agroscope research slaughterhouse after being stunned with CO_2_ and then exsanguinated, scalded, dehaired, and eviscerated. Blood was sampled directly during bleeding after CO_2_ stunning using blood collection tubes with tri-potassium K3EDTA (Vacuette®; Greiner Bio-One GmbH, Kremsmuenster, Austria), which were stored upside down at room temperature for 1 h prior to processing. The Vacuette® tubes were then centrifuged for 15 min at 3000×*g* and subsequently for 2 min at 4000×*g* to isolate plasma. Two aliquots of plasma were stored at − 20 °C in Eppendorf tubes. The liver was removed from each pig carcass and a sample of liver tissue was collected from the half distal part of the right lateral lobe. All samples were individually packed in aluminium foil and immediately snap-frozen in liquid nitrogen. They were further refrigerated at − 80 °C until tissue homogenization for proteomic analysis.

### Label-free quantitative (LFQ) proteomics

**Figure 4:**
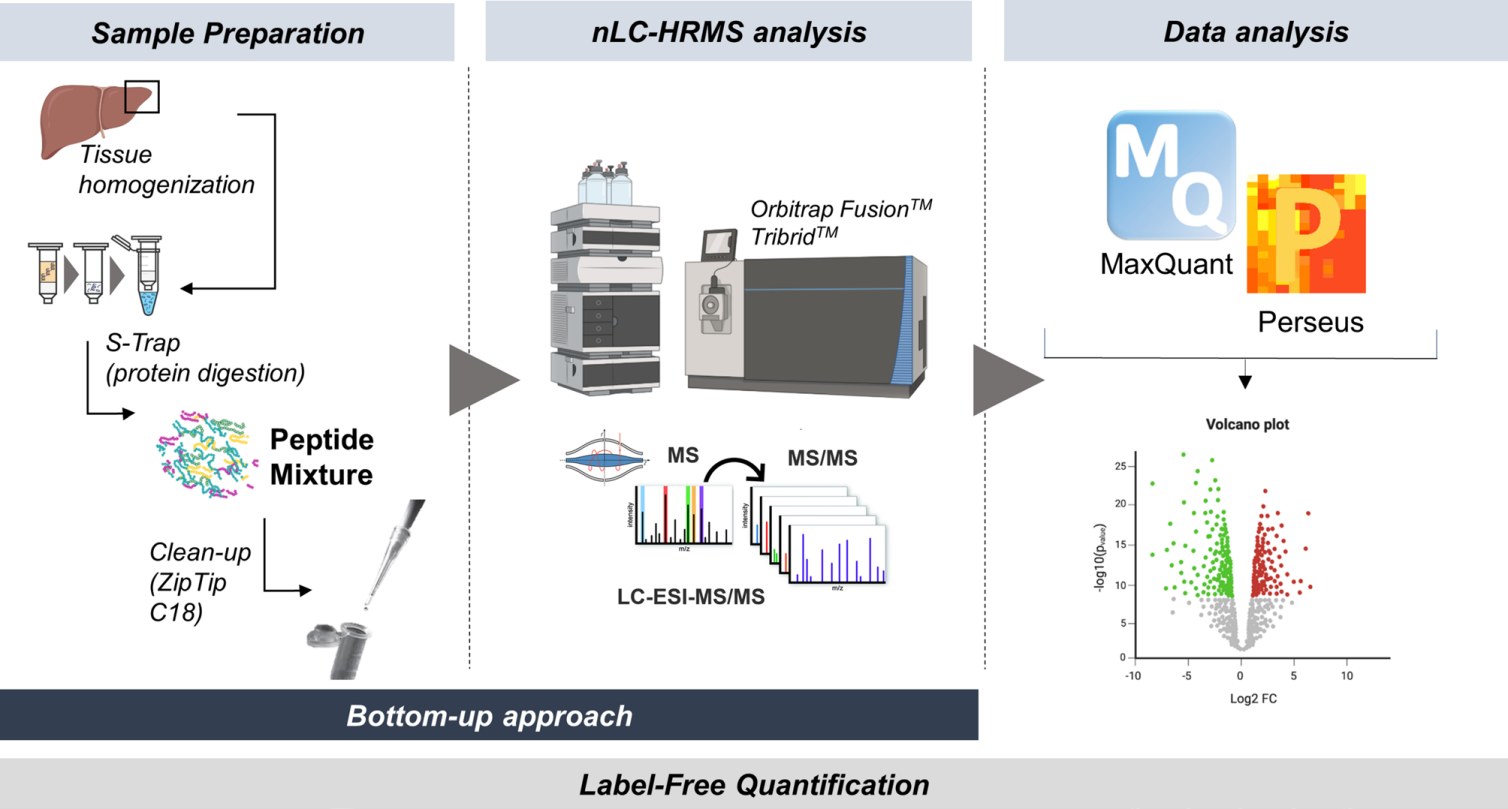
Overview of the experimental workflow applied for the proteomics analysis of pigs’ liver tissue samples.

Here below, a scheme showing the experimental workflow of the label-free quantitative proteomics analysis on liver tissue samples is reported (Fig. 4).

### Sample preparation

#### Tissue homogenization and animal sorting

Two portions for each animal of liver tissue (~ 200 mg each) were randomly collected using a sterile scalpel and transferred into micro-beads (Zirconium Bead 1.5 mm) pre-filled homogenizer tubes, along with 1 mL of lysis buffer (about 20% w/v). The tissue homogenization required a harsh lysis buffer to dissolve hard-to-solubilize proteins, compatible with S-Trap devices used later for proteolysis: SDS 5%, TEAB 50 mM, NaCl 50 mM, MgCl_2_ 5 mM, *Benzonase*® *Nuclease* 500 U/mL, of *cOmplete™ cocktail* (1 tablet/10 mL). A high-energy benchtop homogenizer (BeadBug™ microtube homogenizer), compatible with low working temperatures (4 °C), designed to promote rapid tissue disintegration through constant high-velocity impact of hardened microspheres was used. In detail, 4 cycles of homogenization were conducted at 4000 rpm for 2 min each. Samples were then centrifuged at 10,000 rpm for 30 min at 4 °C, and the supernatant transferred into a new Eppendorf for the protein concentration assessment carried out using the BCA kit.

**Figure 5:**
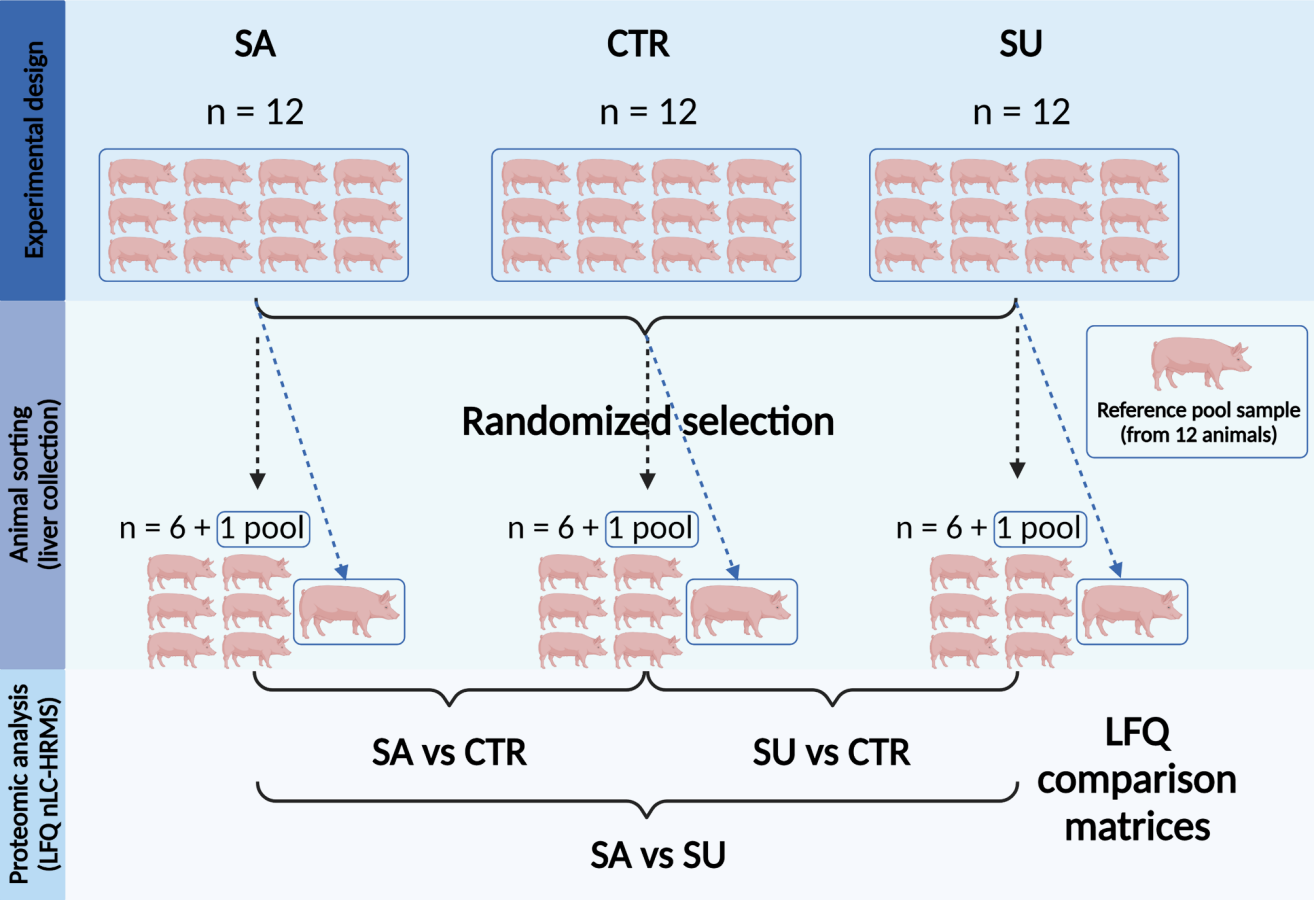
Scheme of the proteomics study design.

Since the label-free quantitative proteomics approach is more prone to errors introduced by measurement conditions than methods involving labeling, one of the main limitations related to the application of label-free methods was the total number of samples that could be analyzed. Therefore, given the impossibility of examining all 36 animals (12 for each experimental group), 6 animals per group were selected following a total randomization approach (Fig. 5). The codes were entered into an open-source algorithm (https://www.random.org/lists/) allowing the elements of a list to be randomly ordered^57^. The first six codes per group were selected for further experiments.

In addition to the 6 selected samples, 3 pools obtained by combining the 12 biological samples from each group, taking the same amount of protein from each (BCA assay measurement), were also prepared and analyzed separately to be used as reference samples for the animal reproducibility evaluation.

### Proteolytic digestion using spin-column S-TRAP

An adequate amount of protein extract (30 μg) was processed following an *in-house* well-optimized protocol for tryptic digestion^58^, based on the usage of S-TRAP spin-columns (Protifi, Huntington, New York, USA) to guarantee excellent yield of enzymatic hydrolysis and efficient removal of reagents or impurities from the digestion process (clean-up process). According to Ferrario et al.^58^, the collected peptic mixtures obtained were then dried under vacuum in the SpeedVac (Eppendorf s.r.l.) at a working temperature of 37 °C, and stored at − 80 °C until analysis.

### nLC-HRMS analysis: Orbitrap fusion tribrid mass spectrometer

The nLC-HRMS (*nano Liquid Chromatography-High Resolution Mass Spectrometry*) method already optimized in our laboratories involves the use of an analytical platform consisting of a Dionex Ultimate 3000 nano-LC chromatograph (Sunnyvale, CA, USA), coupled to the OrbitrapFusion Tribrid high-resolution mass spectrometer (Thermo Scientific, Bremen, Germany). Each sample was injected in triplicate and analyzed as reported by Ferrario et al.^58^. Between each run, stationary phase washes were carried out by injecting 100% ACN to prevent carry-over.

### Proteomics data analysis

The acquired experimental data were firstly processed by means of MaxQuant version 1.6.0 (Max Plank Institute of Biochemistry, Germany). The Andromeda search engine, implemented within the software itself, allowed the identification of proteins by searching a database, namely that of *Sus scrofa* (Taxonomy ID: 9823). The MaxLFQ algorithm (version 1.6.2.3, Max Planck Institute of Biochemistry, Martinsried, Germany) was employed for quantification, considering only so-called unique razor peptides. To ensure accurate identification and quantification of protein mixtures, certain input information regarding the sample preparation method were provided to the software, namely the protease used (trypsin) and the maximum number of missed cleavages (two) allowed. In addition, the match-between-run option was enabled, which was useful for increasing the number of peptides identified and thus the coverage of the proteome in question. A tolerance limit of 5 ppm was set for the identification of precursor ions, consistent with the performance of the high-resolution instrument used. Finally, chemical modifications of some amino acid residues due to sample processing were included, such as the carbamidomethylation of the thiol groups of cysteines as a fixed/static modification, whereas particularly frequent variable modifications were oxidation of methionine and acetylation at the N-terminus of the protein.

Perseus (version 1.6.1. 43; Max Planck Institute of Biochemistry, Martinsried, Germany) was used for facilitating the handling and interpretation of large and complex data matrices derived from MaxQuant, through statistical reworking: LFQ intensity values were first converted to a base-two logarithmic scale and then filtered for significance (Benjamini–Hochberg corrected two-sample t-test with a threshold value of 0.05 False Discovery Rate—FDR). Three comparison matrices were then built for calculating log2 Fold-Change (FC) values (or difference or ratio), indicating the different expression level of individual gene products in the different experimental conditions. As a method of visualizing the results, graphical representations known as Volcano Plots were used, with on the x-axis log2 FC values of a given protein species between two experimental conditions and on the y-axis the -log p-value identifying the significance level of the assignment. Finally, Perseus also allows the reproducibility of biological and technical replicates to be assessed by calculating the Pearson correlation factor. The information about significantly up- and down-regulated proteins was then used to study the modulated pathways in the different experimental groups. Specifically, the String software was used for functional analysis to search for potential interactions between significantly down-regulated (log2 FC < − 0.57) and significantly up-regulated (log2 FC > 0.57) proteins.

### Label-free quantitative peptidomics

#### Experimental design and sample preparation

**Figure 6:**
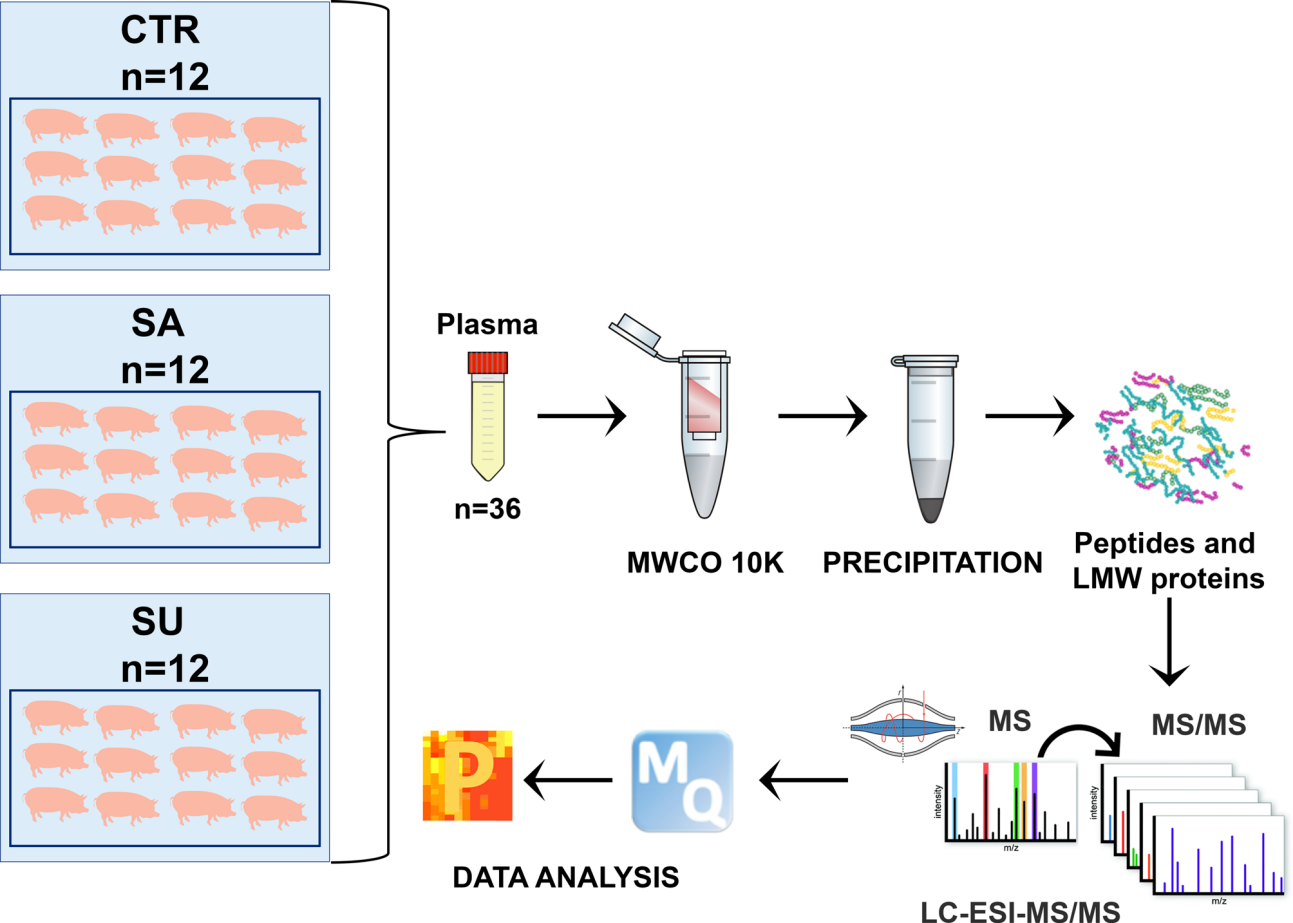
Workflow of the shotgun label-free quantitative peptidomic analysis of peptides on plasma of pigs fed conventional diet (CTR), salty (SA) and sugary (SU) FFP diets.

A shotgun label-free quantitative peptidomic approach was applied to investigate the plasma peptidome of all the thirty-six pigs involved in the study (12 for each experimental group). The workflow included peptide enrichment by ultrafiltration, protein precipitation followed by LC–ESI–MS/MS analysis, as well as identification, label-free quantitation and bioinformatic analysis to identify differential peptides in the three dietary groups. The experimental protocol used in this study is schematically summarized in Fig. 6.

In detail, plasma was isolated from blood samples and then aliquots of 500 µL of plasma were diluted with 32% (v/v) acetic acid and ultra-filtered using Amicon Ultra-0.5 mL centrifugal filters (MWCO 10 K) for high molecular weight protein depletion^59–61^. The flow through was then precipitated with two volumes of cold acetonitrile (ACN) containing 0.1% trifluoroacetic acid (TFA) and centrifuged at 13,200 rpm for 30 min at 4 °C to remove residual proteins. The supernatant containing peptides and low molecular weight proteins was collected, dried, dissolved in 1% (v/v) formic acid and desalted^62^ before mass spectrometric (MS) analysis without any previous digestion.

### Nano-LC–ESI–MS/MS analysis: mass spectrometry-based shotgun peptidomics

Nano-HPLC coupled to MS/MS analysis was performed on all the 36 plasma samples (one for each animal involved in the trial) using a Dionex UltiMate 3000 directly connected to an LTQ Orbitrap Fusion™ Tribrid™ mass spectrometer (Thermo Scientific, Bremen, Germany) equipped with a nano-electrospray ion source, as described for the proteomic approach.

### Peptidomics data analysis

The acquired raw files were subjected to data analysis using MaxQuant software as like as for the proteomics investigation and as described in Toni et al.^63^, setting the enzyme specificity as unspecific^59–61^. The mass spectrometry raw data have been deposited in the ProteomeXchange Consortium (http://proteomecentral.proteomexchange.org) via the PRIDE partner repository with the dataset identifier PXD047855.

Peptides identified by MaxQuant were analyzed by the Perseus software (version 1.5.5.3). Hits to the reverse database were eliminated and the LFQ intensities were converted to a log scale (log2). Only peptides present and quantified in at least 70% biological replicates were considered as positively identified in a group^61^. Principal Component Analysis (PCA) was generated during Perseus analysis, with the resulting datasets. The individual variability of the biological replicates was evaluated by comparing plasma samples belonging to the same experimental group in terms of number of identified peptides, sum of LFQ signal intensity and Pearson correlation coefficient values, calculated using the log2 LFQ intensity.

Specific comparisons were carried out, namely CTR versus SA versus SU, CTR versus SA, CTR versus SU and SA versus SU. A Student’s t-test (FDR ≤ 0.05) was carried out to identify peptides differentially present among the different conditions. Peptides were considered to be differentially present if they were present only in one condition or showed significant t-test difference.

All peptides were searched in the Structurally Annotated Therapeutic Peptides database (SATPdb)^64^ and in the Food-Derived Bioactive Peptides database (DFBP)^65^ to find potentially bioactive peptides. The search was performed applying an “IF” nested function to a matrix that compared the sequence of each peptide found with those of the database (Microsoft Excel 2018, version 16.16.27; Microsoft Corp., Redmond, WA)^60^.

### Supplementary Information

Supplementary Information.

Supplementary Figure S1.

Supplementary Figure S2.

Supplementary Table S1.

## Data Availability

The datasets generated during and/or analysed during the current study are available. Specifically, the mass spectrometry proteomics and peptidomids data have been deposited in the PRIDE partner repository for the ProteomeXchange Consortium with dataset identifiers: PXD049972 (proteomics) and PXD047855 (peptidomics). The account details are available from the corresponding author on reasonable request.
