# Supplementary material for: Preliminary investigation on the impact of salty and sugary former foods on pig liver and plasma profiles using OMICS approaches
Source: Sci Rep. 2024 Aug 21;14:19386. doi: 10.1038/s41598-024-70310-z (PMC11339069; doi:10.1038/s41598-024-70310-z)
Supplement: Supplementary file 3 — Supplementary Figure S2. [file 41598_2024_70310_MOESM3_ESM.pdf]

Impact of dietary inclusion of salty and sugary former food products on the liver and plasma profile of pigs through OMICS approaches

Michele Manoni, Alessandra Altomare, Simona Nonnis, Giulio Ferrario, Sharon Mazzoleni, Marco Tretola, Giuseppe Bee, Gabriella Tedeschi, Giancarlo Aldini, Luciano Pinotti

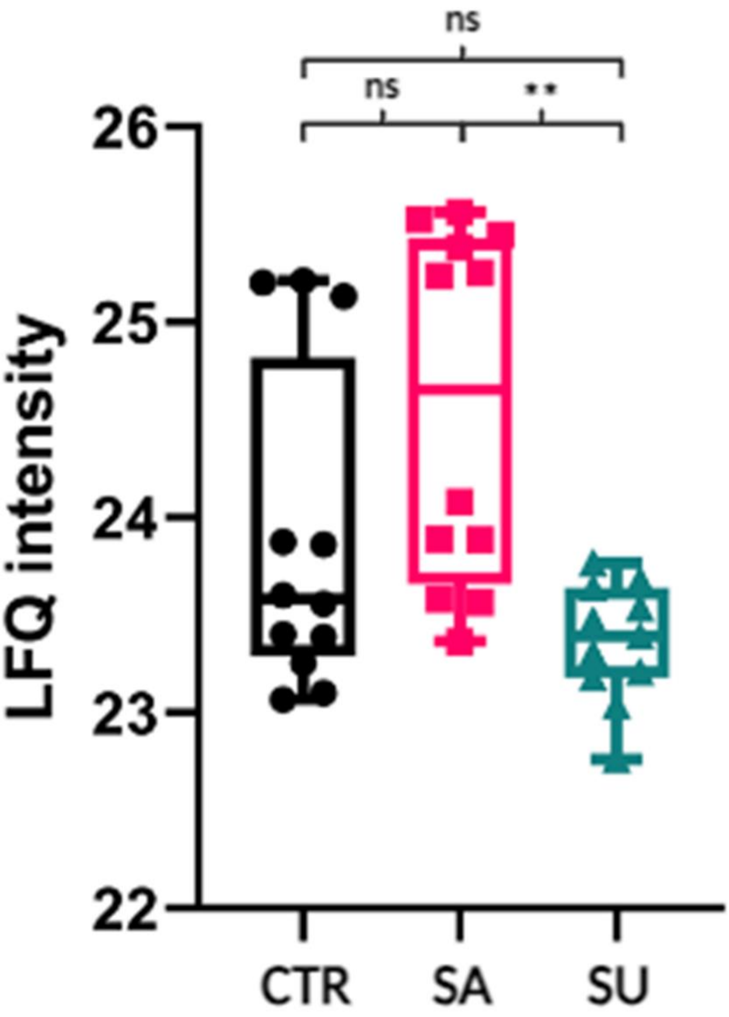

Figure 2. Distribution of LFQ intensity values for KMO in the CTR, SA and SU groups and related significance. ns = non-significant; ** = P < 0.01.
