# Supplementary material for: Preliminary investigation on the impact of salty and sugary former foods on pig liver and plasma profiles using OMICS approaches
Source: Sci Rep. 2024 Aug 21;14:19386. doi: 10.1038/s41598-024-70310-z (PMC11339069; doi:10.1038/s41598-024-70310-z)
Supplement: Supplementary file 4 — Supplementary Table S1. [file 41598_2024_70310_MOESM4_ESM.docx]

**Impact of dietary inclusion of salty and sugary former food products on the liver and plasma profile of pigs through OMICS approaches**

Michele Manoni, Alessandra Altomare, Simona Nonnis, Giulio Ferrario, Sharon Mazzoleni, Marco Tretola, Giuseppe Bee, Gabriella Tedeschi, Giancarlo Aldini, Luciano Pinotti

| **Diet** | **Sample** |  | **Number of identified peptides** | **Variability % (number of identified peptides)** | **LFQ signal intensity** | **Variability % (LFQ signal intensity)** |
| --- | --- | --- | --- | --- | --- | --- |
| CTR | 1 |  | 110 | 8.9 | 4.05E+10 | 29.9 |
|  | 2 |  | 108 | 8.8 | 8.76E+09 | 6.5 |
|  | 3 |  | 54 | 4.4 | 1.42E+09 | 1.0 |
|  | 4 |  | 83 | 6.7 | 2.08E+09 | 1.5 |
|  | 5 |  | 119 | 9.7 | 9.94E+09 | 7.3 |
|  | 6 |  | 108 | 8.8 | 6.22E+09 | 4.6 |
|  | 7 |  | 108 | 8.8 | 1.40E+10 | 10.4 |
|  | 8 |  | 122 | 9.9 | 5.73E+09 | 4.2 |
|  | 9 |  | 140 | 11.4 | 2.46E+10 | 18.2 |
|  | 10 |  | 94 | 7.6 | 6.62E+09 | 4.9 |
|  | 11 |  | 88 | 7.1 | 4.23E+09 | 3.1 |
|  | 12 |  | 98 | 8.0 | 1.11E+10 | 8.2 |
| SA | 1 |  | 132 | 9.1 | 1.57E+10 | 9.5 |
|  | 2 |  | 120 | 8.3 | 3.39E+09 | 2.0 |
|  | 3 |  | 148 | 10.2 | 1.61E+10 | 9.7 |
|  | 4 |  | 142 | 9.8 | 7.86E+09 | 4.7 |
|  | 5 |  | 117 | 8.1 | 6.08E+09 | 3.7 |
|  | 6 |  | 101 | 7.0 | 1.30E+10 | 7.9 |
|  | 7 |  | 131 | 9.1 | 4.94E+09 | 3.0 |
|  | 8 |  | 118 | 8.2 | 2.50E+10 | 15.1 |
|  | 9 |  | 108 | 7.5 | 2.98E+10 | 18.0 |
|  | 10 |  | 109 | 7.5 | 9.36E+09 | 5.6 |
|  | 11 |  | 123 | 8.5 | 2.04E+10 | 12.3 |
|  | 12 |  | 97 | 6.7 | 1.41E+10 | 8.5 |
| SU | 1 |  | 136 | 9.6 | 9.52E+09 | 3.4 |
|  | 2 |  | 140 | 9.9 | 1.15E+10 | 4.1 |
|  | 3 |  | 117 | 8.3 | 8.73E+10 | 31.0 |
|  | 4 |  | 139 | 9.8 | 1.59E+10 | 5.7 |
|  | 5 |  | 88 | 6.2 | 9.78E+09 | 3.5 |
|  | 6 |  | 107 | 7.6 | 8.03E+09 | 2.9 |
|  | 7 |  | 112 | 16.4 | 9.43E+09 | 6.8 |
|  | 8 |  | 115 | 8.1 | 6.11E+09 | 2.2 |
|  | 9 |  | 122 | 8.6 | 6.27E+10 | 22.2 |
|  | 10 |  | 100 | 7.1 | 1.56E+10 | 5.5 |
|  | 11 |  | 99 | 7.0 | 2.59E+10 | 9.2 |
|  | 12 |  | 137 | 9.7 | 2.00E+10 | 7.1 |

**Supplementary Table S1.** Number of identified proteins, sum of the LFQ intensity signal and variance percentage values for each biological replicate belonging to a group (CTR, SA or SU).
